# Operations Research for Pediatric Elective Surgery Planning: Example of a Mathematical Model

**DOI:** 10.3390/bioengineering13020186

**Published:** 2026-02-05

**Authors:** Martina Doneda, Sara Costanzo, Giuliana Carello, Amulya Kumar Saxena, Gloria Pelizzo

**Affiliations:** 1 Department of Information, Management and Production Engineering, University of Bergamo, 24044 Dalmine, Italy; martina.doneda@unibg.it; 2 Pediatric Surgery Department, Buzzi Children’s Hospital, 20154 Milan, Italy; sara.costanzo@asst-fbf-sacco.it; 3 Department of Electronics, Information and Bioengineering, Politecnico di Milano, 20133 Milan, Italy; giuliana.carello@polimi.it; 4 Department of Pediatric Surgery, Chelsea Children’s Hospital, Chelsea and Westminster Hospital NHS Foundation Trust, Imperial College London, London SW10 9NH, UK; amulya.saxena@nhs.net; 5 Department of Biomedical and Clinical Science, University of Milan, 20157 Milan, Italy

**Keywords:** surgery scheduling, pediatric surgery, disruption/restoration, teaching hospital

## Abstract

The management of operating rooms (ORs) is one of the most studied topics in operations research applied to healthcare. In particular, scheduling elective surgeries in a pediatric and teaching hospital can be a challenge because disruptions occur frequently. The aim of our research was to create a mathematical programming model to schedule day hospital (DH) patients, considering possible disruptions and defining how to best manage the rescheduling process. Our study originates from a collaboration between a high-volume pediatric surgery department and operations research practitioners. The possible disruptions we consider are emergencies and same-day cancellations of planned hospital operations. Elective DH surgeries are scheduled considering the waiting list and the patients’ clinical priorities, generating a nominal schedule. This schedule is optimized in conjunction with a series of back-up schedules to guarantee that OR activity immediately recovers in case of a disruption. An ILP-based approach to the problem is proposed. We enumerate a representative subset of the possible emergency and no-show scenarios, and for each of them a back-up plan is designed. The approach reschedules patients, minimizing disruptions with respect to the nominal schedule, and applies an as-soon-as-possible policy in case of emergencies to ensure that all patients receive timely care. The approach is shown to be effective in managing disruptions, ensuring that the waiting list is managed properly, with a balanced mix of urgent and less urgent patients. It provides an effective solution for scheduling patients in a pediatric hospital, considering the unique features of such facilities.

## 1. Introduction

Operations research—or decision science—is a branch of applied mathematics that deals with the optimization of systems and processes, and operating room (OR) management is one of the most studied topics in its application to healthcare. However, there are still unaddressed specific challenges concerning surgery planning in both pediatric settings and teaching hospital ones.

A teaching hospital (TH) is a hospital that cooperates with medical and nursing schools, education programs and research centers to improve healthcare through learning and research. These institutions provide medical care and community care, train professionals for the future of health, stimulate the application of technological innovations, carry out translational research, and establish international collaborations for a better quality of care. The functioning of ORs in THs is different from that in other settings where training and research do not play such a preponderant role, as surgical times can be longer and costs higher for staff training and technology dissemination, and data collection can be especially difficult [1]. In particular, the activities of a pediatric surgical OR in a TH present specific criticalities, regarding which it is important to recall that (i) the high surgical activity and the concentration of many specialties oftentimes prevents the possibility of dedicating an OR to emergencies [2] and that (ii) that there is a much higher rate of cancellations on the scheduled day [3] compared to adult patients for rather unpredictable reasons, such as sudden-onset upper respiratory tract infections, exanthematous diseases, and the incidence of complications in fragile or complex patients, which may make it necessary to postpone an intervention. The reasons for the suspension or cancellation of activities are multifactorial and may depend on the patients, the families, the surgeons, the anesthetists, the OR staff, and the availability of postoperative services [4]. According to internationally recognized recommendations, the incidence of pediatric elective surgery cancellations on the scheduled day should remain below 5% [5]. Unfortunately, the limit of 5% cancellations on the scheduled day is often exceeded, thus resulting in an enormous waste of resources, particularly without an effective correction system dedicated to the pediatric area.

In recent years, the management of pediatric surgeries has steered towards an organizational approach relying more and more on day hospital (DH) procedures. Indeed, a DH approach is increasingly being adopted not only for minor surgery, but also for major interventions, as is already the case in some pediatric contexts [6]. The use of DHs allows for optimization not only from an economic point of view, for instance, leading to savings on hospitalization costs and personnel employed, but also in terms of patient satisfaction. However, even such a streamlined approach requires proper managerial tools to manage disruptions and use resources effectively, particularly in a TH where resources are extremely scarce.

To this end, the research question that guided us was how to best schedule DH patients given a waiting list and a certain surgical capacity, while considering possible disruptions to the scheduling and, if something does interrupt the plan, how to best manage the rescheduling process. To answer this question, we created an ILP-based approach that would guide the cancellation and rescheduling process, minimizing the disruption and related costs [7].

The remainder of the paper is organized as follows: Section 2 reviews the relevant literature about operations research applied to OR management so as to position our contribution, which is presented in full form in Section 3. Section 4 presents some computational results of its usage in a set of synthetic instances, and in Section Case Study an applicative case study can be found. Insights and limitations are discussed in Section 5, and Section 6 concludes the paper.

## 2. Literature

There is a vast amount of operations research literature on OR management. This is evident from the numerous surveys published in the early 2010s [8,9] and even in more recent years [10,11]. In these surveys, the literature is usually classified according to the decision level and planning horizon addressed:

1. In the long term, the strategic-level decisions, such as the number of ORs and staff size, are addressed [12];
2. Medium-term decisions, such as OR allocation to specialties, are addressed at the tactical level [13,14];
3. Short-term decisions are addressed at the operational level: advance scheduling assigns each patient to a day and OR, while allocation scheduling determines the sequence of surgeries in each OR and day [15].

In the present paper we consider the operational level. The capacity of ORs is shared among surgical teams, and an open scheduling policy is applied [16]. We address both advance and allocation scheduling for DH patients under the assumption that uncertain events may happen. Further, as this work deals with DH patients, we do not take into consideration the literature explicitly dealing with coordination with upstream or downstream resources.

The most commonly considered source of uncertainty when planning OR activity is related to surgery times. This issue has been addressed in many papers, particularly at the operational level. The main approaches are based on stochastic programming [17,18,19], robust optimization [20,21,22], and distributionally robust optimization [23]. However, these approaches may result in overly conservative solutions. This drawback is even more relevant when considering inherently unpredictable events, such as no-shows (i.e., last-minute cancellations of planned operations) or emergencies (i.e., external inputs that may impact the planned schedule by delaying subsequent operations and occupying OR capacity). Such disruptions may cause significant inconvenience and distress to patients and, in the case of pediatric patients, their families [24,25,26].

Many strategies have been proposed to tackle emergencies. In some works, it is suggested to devote one or more ORs to the management of any emergency that may occur [27]. However, this configuration can become undesirable when there are few ORs and/or a relatively small number of emergencies. Other works suggest sharing OR capacity between elective surgeries and emergencies. This is mainly achieved in three ways [28]: (i) reserving some OR capacity to accommodate emergencies [29]; (ii) not explicitly reserving any capacity for emergencies, but proactively scheduling elective procedures in a manner that facilitates emergency management; and (iii) designing a set of policies that can be deployed to accommodate emergencies in an online fashion. Some noteworthy works suggest strategies for managing emergencies without explicitly dedicating OR time to them. For instance, Lamiri et al. [30] use a stochastic model to schedule elective patients in such a way as to minimize OR overtime costs linked to emergencies. Instead, Essen et al. [31] propose spreading the completion times of elective surgeries as much as possible, so that emergencies can be accommodated promptly. In [32], the daily schedule is assumed as given and the problem of inserting emergencies is considered. Alternative actions to accommodate emergencies are evaluated based on waiting-time targets for emergencies, adherence to the original elective surgery plan and possible overtime. To generate a schedule that can react well to emergencies and treat them quickly, the authors of [33] propose an ILP model that leverages on reserving spare capacity to keep the probability of excessive workload below a threshold. Reactive scheduling has been studied as well. In [34], the authors propose the minimization of a numerical indicator named Total Expected Disturbance, calculated using historical data on emergencies and estimates of the probability of a given emergency occurring in a certain moment. In [35], an approach is proposed to guarantee that each emergency, whenever it may occur, can be treated within 20 min of its happening. An ILP model of the integrated OR planning and scheduling problem is formulated: it allocates slack time and sets constraints on the waiting-time targets for emergencies.

In contrast with the study of emergency management, the handling of no-shows has received less attention in the operations research literature.

Dantas et al. [36] provide a comprehensive review of no-show appointments in healthcare, not exclusively focused on surgeries, to trace the main determinants that drive the phenomenon. Still not in the strict domain of surgical specialties, Zacharias and Pinedo [37] propose a scheduling policy in which no-shows are counterbalanced by overbooking. Berg et al. [38] consider individually tailored no-show probabilities and levers for patient ordering, reducing the expected discomfort of patients by placing the ones who are more likely to be no-shows towards the end of the patient sequence if possible. M’Hallah and Visintin [39] use a stochastic model to schedule patients, assuming that cancellations due to no-shows can be represented with a random variable. As pointed out in [40], the availability of data on no-shows may be limited because it is hard to determine if a patient will not show up for an appointment that has not taken place yet, and the authors resort to simulations to test the impact of no-shows on their scheduling model. Salazar et al. [41], like in other works that employ machine learning techniques to predict no-shows for appointments, propose a novel classifier, which, although it is very well performing, is hardly generalizable as it was built with data from a specific non-surgical context.

This work contributes to the literature on pediatric elective surgical scheduling by providing an approach to managing emergencies and no-shows with a disruption/restoration perspective. Comparing and contrasting it with other robust approaches found in the literature, our approach allows us to guarantee a prompt recovery in whatever case may occur while guaranteeing good performance regarding the nominal schedule, as no slack time is required. Indeed, this is done without predicting possible scenarios or leaving spare capacity to allow for some planning flexibility. A proactive plan is built that is able to accommodate emergencies and react to no-shows. Rather than managing emergencies online, ready-to-deploy alternative schedules are built alongside the nominal one. The horizon considered spans several days, allowing the reorganization, if needed, of the surgeries assigned to successive days. We would like to highlight that, to the best of our knowledge, no approach in the literature incorporates the generation of back-up plans along with the nominal one in surgery scheduling.

## 3. Materials and Methods

We use an approach based on the concept of disruption/restoration, often applied in the field of telecommunications and transportation planning. A waiting list is considered and the operations of the DH patients on the list are planned, generating a schedule to be followed in the absence of interruptions (nominal schedule, NS). The waiting list is created from a group of “ideal patients”, synthetically derived from examples of pathologies that typically fall within the DH paradigm. The NS is generated in a resilient way so that it can be easily readjusted in the event of any interruptions. In fact, together with the NS, restoration (or back-up) schedules (RSs) are also generated, one for each interruption considered, which are applied if the interruption occurs, thus allowing a rapid restoration of operations while maintaining good performance regarding the nominal schedule. Both the NS and the RSs are optimized based on the needs of the patients and the department. The interruptions to the surgical schedule that we consider are of two types:

1. Emergencies, modeled as external inputs that can impact the planned schedule, delaying subsequent operations and occupying the capacity of the OR;
2. No-shows, defined as last-minute cancellations of planned operations.

The tool used to generate the schedule is a mathematical optimization approach that considers the following data:

1. Characteristics of the patients on the waiting list: time spent on the list, surgical urgency, estimated duration of the operation, and compatible teams;
2. Characteristics of the department (teaching hospital): surgical capacity and the availability of the different teams.

The output is the DH surgical schedule, which assigns the patients to a day and an OR and defines the order of the patients assigned to the same day and OR and, consequently, the start and end time of each surgical operation. Based on this, the occupancy of the OR is defined and, with this plan, at any given moment it is possible to determine whether or not an OR is empty or what its first availability is if a patient is undergoing surgery.

The optimization model considers the aforementioned interruptions: possible emergencies and possible no-shows. In the optimization process it is assumed that only one emergency or no-show can occur on any given day. These assumptions represent the core of the work and have been face-validated by the clinical staff of a pediatric ward in a teaching hospital. The lack of consolidated historical data on these issues prevented us from better modeling the uncertainty set of this scheduling problem, and thus scenario enumeration represents a valuable alternative when no further information can be obtained.

The emergency problem is two-faceted: on the one hand, determining which OR to assign each emergency that might occur and, on the other hand, determining how the schedule should be modified to accommodate the disruption. The no-show problem instead deals with rescheduling the disruptive patient and adjusting the remainder of the schedule. The optimization problem considers the following decisions: On which day is a patient scheduled? In which order are patients scheduled for each day? To which OR is an emergency assigned? How to recover in case of emergency and no-shows (alternative assignments to days and alternative schedules)?

The model represents the above decisions with decision variables. By solving the model, we assign a value to each decision variable, thus providing an answer to the aforementioned decisions. As mentioned, a solution of the model provides both an NS and a set of RSs, one for each considered disruption. The NS and the RSs must be acceptable, that is, they must meet some requirements. The model is a mathematical representation of the decision problem of generating acceptable schedules, and, among them, it selects the one that optimizes relevant metrics. Requirements are described by mathematical expressions (constraints), which we group in blocks, according to their scope.

Let us now move on to the formal description of the mathematical model of the problem. The primary aim is to assign patients (belonging to a set, I) to a set of ORs, J, over a planning horizon, D, minimizing the penalty associated with (not) assigning them and the time they spend on the list, both weighted by their surgical priority. Secondarily, it is necessary to design a feasible RS schedule for each considered disruption that is as close to the nominal one as possible.

Some information is given about each patient, i, in the DH waiting list, I. First, each patient’s surgical deadline, $l_{i}$, is known. This represents the clinically recommended maximum amount of time that should be allowed to elapse between being inserted into the list and the day of the surgery [7]. From this deadline, it is possible to determine each patient’s urgency coefficient, $u_{i}$, equal to $360/l_{i}$ for each i∈I. Secondly, it is known how many days patient i has already spent on the waiting list, expressed by parameter $m_{i}$. In order to determine the total waiting time for each patient, it is possible to calculate for each day, d, of the planning horizon a penalty factor associated with scheduling them on a given $d\left(p_{id}\right)$. We also determine a penalty factor, $q_{i}$, associated with not scheduling patient i in the planning period:

(1) $$p_{id}=\left[d+\max\left\{m_{i}+d-l_{i};0\right\}\right]u_{i}\;\;\;\;\;\;\;\;\;\;\;\;\;\;\;\;\;\;\;\;\;\;\;\;\forall i\in I,d\in D$$

(2) $$q_{i}=\left[\left(m_{i}+|D|+1\right)+max\left\{\left(m_{i}+|D|+1-l_{i}\right);0\right\}\right]u_{i}\;\;\;\;\;\;\;\;\;\;\;\;\;\;\;\;\;\;\;\;\;\;\;\;\forall i\in I$$

As previously mentioned, the aim of the problem is to assign patients to a schedule in such a way that the penalty of (not) assigning them is as small as possible. We introduce a binary decision variable, $x_{idj}$, which equals 1 when patient i is assigned to ORj on day d, and formulate the following objective function, which was originally introduced in [21]:

(3) $$min\sum_{i\in I}\sum_{j\in J}\left\{\sum_{d\in D}p_{id}x_{idj}+q_{i}\sum_{d\in D}\left(1-x_{idj}\right)\right\}$$

The objective function, besides reducing patients’ tardiness, forces patients to be operated on as soon as possible, as the number of days before surgery is weighted in the $p_{id}$ term as well.

Let us now move on to the description of constraints. For clarity, they will be divided into blocks. A lookup table providing a summary of all sets and parameters used in the model is presented in Table 1, together with the decision variables and their domains. This table contains all entities referenced in the equations from (1) to (49). It is worth remarking that in this model time is not continuous, but rather it is represented by a set of time slots or moments. All time-related parameters and decision variables used in the following formulations are expressed as numbers of time slots.

Block A consists of patient assignment constraints:

(4) $$\sum_{d\in D}\;\sum_{j\in J}x_{idj}\leq 1\;\;\;\;\;\;\;\;\;\;\;\;\;\;\;\;\;\;\;\;\;\;\;\;\forall i\in I$$

(5) $$x_{idj}\leq a_{idj}\;\;\;\;\;\;\;\;\;\;\;\;\;\;\;\;\;\;\;\;\;\;\;\;\;\;\forall i\in I,j\in J,d\in D$$

(6) $$\sum_{i\in I}t_{idj}x_{idj}\leq T_{dj}\;\;\;\;\;\;\;\;\;\;\;\;\;\;\;\;\;\;\;\;\;\;\;\;\;\forall j\in J,d\in D$$

Constraints (4) make sure that each patient is scheduled at most once. Parameter $a_{idj}$ describes the compatibility between patients, days and ORs: it is equal to 1 if a surgical team can operate on patient i on day d and OR j. Constraints (5) ensure that only the proper patient–day–OR triples can be considered for assignment. Constraints (6) make sure that the sum of the surgical times, $t_{idj}$, for all patients, i, assigned to OR j on day d does not exceed the total OR capacity, $T_{dj}$.

Block B contains patient ordering constraints, which assign each scheduled patient to a position in the schedule. The possible positions are represented by a set, S. The maximum number of positions required for any possible plan is calculated as:

(7) $$|S|=\left\lceil \underset{d\in D,j\in J}{max}T_{dj}/\underset{i\in I,d\in D,j\in J}{min}t_{idj}\right\rceil$$

Ordering is enforced only on a set W⊂D of days, the first upcoming day(s) of the planning period, for which we want to guarantee RS plans. We introduce three other decision variables. First, $y_{igjs}$ is a binary decision variable equal to 1 if patient i is assigned to OR j on day g in position s∈S. The precedence variable, $\nu_{irgj}$, is a binary variable equal to 1 if patient i is assigned after patient r on g,j.

Based on the order, the integer variable, $\xi_{igj}$, the starting time for i’s surgery on g,j can be computed.

(8) $$\sum_{s\in S}y_{igjs}=x_{igj}\;\;\;\;\;\;\;\;\;\;\;\;\;\;\;\;\;\;\;\;\;\;\;\;\forall i\in I,j\in J,g\in W$$

(9) $$\sum_{i\in I}y_{igj\left(s+1\right)}\leq \sum_{i\in I}y_{igjs}\;\;\;\;\;\;\;\;\;\;\;\;\;\;\;\;\;\;\;\;\;\;\;\;\;\forall j\in J,g\in W,s\in S\setminus \left\{\left|S\right|\right\}$$

(10) $$\sum_{i\in I}y_{igjs}\leq 1\;\;\;\;\;\;\;\;\;\;\;\;\;\;\;\;\;\;\;\;\;\;\;\;\;\;\forall j\in J,g\in W,s\in S$$

(11) $$\xi_{igj}=\sum_{r\in I:r\neq i}t_{rgj}\cdot \nu_{irgj}+x_{igj}\;\;\;\;\;\;\;\;\;\;\;\;\;\;\;\;\;\;\;\;\;\;\;\;\;\;\;\forall i\in I,g\in W,j\in J$$

(12) $$y_{igjs}+\sum_{n\in S:n<s}y_{rgjn}-1\leq \nu_{irgj}\;\;\;\;\;\;\;\;\;\;\;\;\;\;\;\;\;\;\;\;\;\;\;\;\;\forall i,r\in I:r\neq i,g\in W,j\in J,s\in S$$

(13) $$\nu_{irgj}\leq x_{rgj}\;\;\;\;\;\;\;\;\;\;\;\;\;\;\;\;\;\;\;\;\;\;\;\;\forall i,r\in I,g\in W,j\in J$$

(14) $$\nu_{irgj}+\nu_{rigj}\leq 1\;\;\;\;\;\;\;\;\;\;\;\;\;\;\;\;\;\;\;\;\;\;\;\;\forall i,r\in I:r\neq i,g\in W,j\in J$$

(15) $$\xi_{igj}\leq \left(T_{gj}+1-t_{igj}\right)\cdot x_{igj}\;\;\;\;\;\;\;\;\;\;\;\;\;\;\;\;\;\;\;\;\;\;\;\;\;\forall i\in I,g\in W,j\in J$$

Constraints (8) link variables $y_{igjs}$ and $x_{igj}$, making sure that one and only one position is assigned to each scheduled patient. Constraints (9) guarantee that a position can be assigned only if the previous one is. Constraints (10) ensure that each position is assigned at most once. Constraints (11) calculate the starting time of each scheduled surgery. Constraints (12)–(14) calculate the precedence variable, $\nu_{irgj}$. Constraints (15) force the starting time of i on day g and OR j to be 0 if patient i is not scheduled on g,j.

Blocks A and B determine the NS and its order. The following four blocks of constraints deal with the management of emergencies.

In order to manage an emergency, it is important to know what the current status of each OR is in the moment at which the emergency occurs. We define a non-negative variable, $C_{gj}$, that encodes the slot, h, in which OR j will be released after the end of the last surgery. Complementarily, $\rho_{hgj}$ is a binary variable equal to 1 if OR j on day g is empty in time slot h. If the OR is not empty, binary decision variables, $\lambda_{igjh}$, are used to identify the patient, i, that is currently being operated on in each OR,j, at h,g. Knowing whether the OR is empty or not, and, in the latter case, who is the patient currently being operated on, allows us to determine the quantity, $\Xi_{hgj}$, the first availability of OR j after moment h. To calculate the values of these variables, we introduce a series of constraints related to ORs, Block C:

(16) $$C_{gj}\geq \xi_{igj}+t_{igj}x_{igj}\;\;\;\;\;\;\;\;\;\;\;\;\;\;\;\;\;\;\;\;\;\;\;\;\;\;\forall i\in I,j\in J,g\in W$$

(17) $$h-C_{gj}\leq \left|H\right|\rho_{hgj}\;\;\;\;\;\;\;\;\;\;\;\;\;\;\;\;\;\;\;\;\;\;\;\;\;\;\forall g\in W,h\in H,j\in J$$

(18) $$h-C_{gj}\geq \rho_{hgj}-\left|H\right|\left(1-\rho_{hgj}\right)-1\;\;\;\;\;\;\;\;\;\;\;\;\;\;\;\;\;\;\;\;\;\;\;\;\forall g\in W,h\in H,j\in J$$

(19) $$\sum_{i\in I}\lambda_{igjh}=1-\rho_{hgj}\;\;\;\;\;\;\;\;\;\;\;\;\;\;\;\;\;\;\;\;\;\;\;\;\forall g\in W,j\in J,h\in H$$

(20) $$\xi_{igj}+t_{igj}x_{igj}\geq h\cdot \lambda_{igjh}\;\;\;\;\;\;\;\;\;\;\;\;\;\;\;\;\;\;\;\;\;\;\;\;\;\forall i\in I,g\in W,j\in J,h\in H:h\neq 0$$

(21) $$\xi_{igj}\leq h\cdot \lambda_{igjh}+\left|H\right|\left(1-\lambda_{igjh}\right)\;\;\;\;\;\;\;\;\;\;\;\;\;\;\;\;\;\;\;\;\;\;\;\;\;\forall i\in I,g\in W,j\in J,h\in H:h\neq 0$$

(22) $$\Xi_{gjh}\geq \xi_{igj}+t_{igj}x_{igj}-\left|H\right|\left(1-\lambda_{igjh}\right)\;\;\;\;\;\;\;\;\;\;\;\;\;\;\;\;\;\;\;\;\;\;\;\;\forall i\in I,g\in W,j\in J,h\in H$$

(23) $$\Xi_{gjh}\leq \xi_{igj}+t_{igj}x_{igj}+\left|H\right|\left(1-\lambda_{igjh}\right)\;\;\;\;\;\;\;\;\;\;\;\;\;\;\;\;\;\;\;\;\;\;\;\;\;\forall i\in I,g\in W,j\in J,h\in H$$

Constraints (16)–(19) serve the purpose of calculating the time at which each OR becomes available after having completed all assigned surgeries and identifying which patients are being operated on in each OR, j, at each moment, h, respectively. For each moment (h), OR (j) and day (g), Constraints (20) and (21) link the start and end time of patient i’s surgery with the variable $\lambda_{igjh}$. Constraints (22) and (23) calculate what is the first availability of OR j on day g, starting from moment h.

An emergency that occurs in time slot h of day g must be managed and assigned to an OR: a binary variable, $\eta_{hdj}$, is defined to determine which OR the emergency that occurs at moment h is assigned to. The emergency assignment constraints of Block D guarantee that each emergency is properly managed:

(24) $$\sum_{j\in J}\eta_{hdj}=1\;\;\;\;\;\;\;\;\;\;\;\;\;\;\;\;\;\;\;\;\;\;\;\;\forall h\in H,d\in W$$

(25) $$\Xi_{gjh}\leq \Xi_{gkh}+\left|H\right|\left(1-\eta_{hgj}\right)\;\;\;\;\;\;\;\;\;\;\;\;\;\;\;\;\;\;\;\;\;\;\;\;\forall g\in W,j\in J,k\in J,h\in H$$

Constraints 24 force the assignment of each possible emergency to exactly one OR. Constraints 25 force the emergency to be assigned to the first available OR.

Block E contains constraints used to evaluate the impact of emergencies on scheduled patients. A new concept is introduced: emergency length class. As the length of an emergency cannot be known in advance, it is assumed to belong to a predetermined class that approximates it. The set of possible emergency lengths is defined as L. Together with h (the moment the emergency occurs) and g (the day), the triplet h,g,l describes a specific scenario: an emergency of class l∈L occurring on day g in time slot h. The impact is expressed using the binary variable $\chi_{igj}$, which is equal to 1 if patient i is impacted by the assignment of an emergency to OR j, that is, if the surgery of i in the NS is scheduled to begin after h. The RSs are defined by binary assignment variables, ${\bar{x}}_{hglidj}$. For each scenario encoded by h,g,l, they store the information regarding its associated RS. Binary variables, $\mu_{hgli}$, account for patients who cannot be rescheduled in the current planning period.

(26) $$\xi_{igj}=\sum_{r\in I:r\neq i}t_{rgj}\cdot \nu_{irgj}+x_{igj}\;\;\;\;\;\;\;\;\;\;\;\;\;\;\;\;\;\;\;\;\;\;\;\;\;\forall i\in I,g\in W,h\in H$$

(27) $$\sum_{j\in J}\xi_{igj}\geq h\cdot \chi_{hgi}+\varepsilon \cdot \chi_{hgi}\;\;\;\;\;\;\;\;\;\;\;\;\;\;\;\;\;\;\;\;\;\;\;\;\forall i\in I,g\in W,h\in H$$

(28) $$\sum_{d\in D:d<g+\Delta }\;\sum_{j\in J}{\;\bar{x}}_{hglidj}\geq \chi_{hgi}\;\;\;\;\;\;\;\;\;\;\;\;\;\;\;\;\;\;\;\;\;\;\;\;\;\forall i\in I,h\in H,g\in W,l\in L$$

(29) $$\sum_{k\in D:k\geq d;k\leq d+\Delta }\;\sum_{j\in J}{\bar{x}}_{hglikj}\geq \sum_{j\in J}x_{idj}-\mu_{hgli}\;\;\;\;\;\;\;\;\;\;\;\;\;\;\;\;\;\;\;\;\;\forall h\in H,g\in W,i\in I,d\in D,l\in L$$

Constraints (26) and (27) force the suitable value of the variable $\chi_{igj}$. In (27), ε is a sufficiently small constant. Constraints (28) force each impacted patient to be considered in the rescheduling, while Constraints (29) determins if and how many patients are excluded ($\mu_{hgli}$) from the rescheduling.

Then, we introduce Block F, which contains the constraints related to the rescheduling caused by the emergency:

(30) $$\sum_{j\in J}x_{idj}+\sum_{j\in J}\sum_{k\in D:k<d}{\bar{x}}_{hglikj}\leq 1\;\;\;\;\;\;\;\;\;\;\;\;\;\;\;\;\;\;\;\;\;\;\;\;\;\forall i\in I,d\in D,h\in H,g\in W,l\in L$$

(31) $$\sum_{j\in J}\sum_{k\in D}{\bar{x}}_{hglikj}\leq \sum_{j\in J}\sum_{d\in D}x_{idj}\;\;\;\;\;\;\;\;\;\;\;\;\;\;\;\;\;\;\;\;\;\;\;\;\;\;\forall h\in H,g\in W,l\in L,i\in I$$

(32) $${\bar{x}}_{hgligj}\geq x_{igj}-\chi_{hgi}\;\;\;\;\;\;\;\;\;\;\;\;\;\;\;\;\;\;\;\;\;\;\;\;\forall h\in H,g\in W,i\in I,j\in J,l\in L$$

(33) $$\sum_{d\in D}\sum_{j\in J}{\bar{x}}_{hglidj}\leq 1\;\;\;\;\;\;\;\;\;\;\;\;\;\;\;\;\;\;\;\;\;\;\;\;\forall h\in H,g\in W,i\in I,l\in L$$

(34) $${\bar{x}}_{hglidj}\leq a_{idj}\;\;\;\;\;\;\;\;\;\;\;\;\;\;\;\;\;\;\;\;\;\;\;\;\forall h\in H,g\in W,i\in I,j\in J,d\in D,l\in L$$

(35) $$\sum_{i\in I}t_{idj}{\bar{x}}_{hglidj}\leq T_{dj}\;\;\;\;\;\;\;\;\;\;\;\;\;\;\;\;\;\;\;\;\;\;\;\;\forall j\in J,\;h\in H,d\in W,l\in L:d>g$$

(36) $$\sum_{i\in I}t_{igj}{\bar{x}}_{hgligj}\leq T_{gj}-min\left\{\gamma_{l};\left(T_{gj}-h\right)\right\}\cdot \eta_{hgj}+\Omega \;\;\;\;\;\;\;\;\;\;\;\;\;\;\;\;\;\;\;\;\;\;\;\;\forall j\in H,g\in W,l\in L$$

Constraints (30) prevent assignments being anticipated with respect to their original date. Constraints (31) prevent patients who were not previously included in the schedule being included in the new schedule. Constraints (32) maintain the original assignment of patients who have not been affected by the emergency considered in the scenario. Constraints (33)–(35) correspond to Constraints (4)–(6) for the nominal assignment problem, while Constraints (36) allow for some overtime (defined by parameter Ω) to be accepted on the day of the emergency, as the emergency itself occupies some OR time. This is the last block of constraints concerning emergency management, the following blocks dealing instead with the management of no-shows.

The number of possible no-show scenarios is enumerable by definition. Only patients that have been assigned in the NS to g∈W can become no-shows, so, different from what we did for the modeling of emergency constraints, it is not necessary to make any additional assumptions. To address no-shows, we introduce the concept of a surrogate (or substitute) patient, a patient who is nominally assigned to the day after the emergency occurs and who can be called to fill the gap left by the no-show. The operating assumption here is that the selected surrogate patient has been made aware of the possibility and can therefore be summoned if needed. The no-show patient is then rescheduled after a predetermined number of days (or within a maximum number of days). Similarly, for each no-show scenario, the rest of the schedule is reset to accommodate the disruption. The RSs are described by binary assignment variables, ${\hat{x}}_{\mathrm{bgidj}\;}$. For each scenario encoded by no-show patient b on day g, the information regarding its associated RS is stored. Block G defines a substitute patient for all ORs, who is identified by the binary variable $\theta_{igj}$:

(37) $$\sum_{i\in I}\theta_{ijg}=1\;\;\;\;\;\;\;\;\;\;\;\;\;\;\;\;\;\;\;\;\;\;\;\;\forall j\in J,g\in W$$

(38) $$\sum_{j\in J}\theta_{ijg}\leq \sum_{k\in J}x_{i(g+1)k}\;\;\;\;\;\;\;\;\;\;\;\;\;\;\;\;\;\;\;\;\;\;\;\;\forall i\in I,g\in W$$

(39) $$\theta_{ijg}\leq a_{ijg}\;\;\;\;\;\;\;\;\;\;\;\;\;\;\;\;\;\;\;\;\;\;\;\;\forall g\in W,j\in J,i\in I$$

(40) $${\hat{x}}_{bgigj}\geq \theta_{igj}+x_{bgj}-1\;\;\;\;\;\;\;\;\;\;\;\;\;\;\;\;\;\;\;\;\;\;\;\;\forall b\in I,g\in W,i\in I,j\in J$$

(41) $$\sum_{j\in J}{\hat{x}}_{bgb(g+\hat{\Delta })j}\geq \sum_{j\in J}x_{bgj}\;\;\;\;\;\;\;\;\;\;\;\;\;\;\;\;\;\;\;\;\;\;\;\;\forall b\in I,g\in W,i\in I$$

Constraints (37) force the unique substitute patient for each OR to be unique. Constraints (38) force the substitute patient to be a patient originally scheduled for the day after g∈W. Constraints (39) ensure that the substitute patient is compatible with the OR they may be called to cover. Constraints (40) make sure that the substitute patient is scheduled instead of the patient they are called to cover for, in the scenario in which the latter is a no-show. The constraints ensure that the no-show patient is rescheduled after $\hat{\Delta }$ days. Constraints (41) can be substituted with the following Constraints (42), in case we do not want to fix the delay but just want to ensure that the no-show patient is rescheduled within Δ days:

(42) $$\sum_{j\in J}\sum_{d\in D:d<g+\Delta }{\hat{x}}_{bgbdj}\geq \sum_{j\in J}x_{bgj}\;\;\;\;\;\;\;\;\;\;\;\;\;\;\;\;\;\;\;\;\;\;\;\;\forall b\in I,g\in W,i\in I$$

Block H deals with back-up plans in case of reassignment whenever a no-show occurs:

(43) $$\sum_{d\in D}\sum_{j\in J}{\hat{x}}_{bgidj}\leq 1\;\;\;\;\;\;\;\;\;\;\;\;\;\;\;\;\;\;\;\;\;\;\;\;\;b\in I,g\in W,i\in I$$

(44) $${\hat{x}}_{bgidj}\leq a_{idj}\;\;\;\;\;\;\;\;\;\;\;\;\;\;\;\;\;\;\;\;\;\;\;\;\forall h\in H,g\in W,i\in I,j\in J,d\in D$$

(45) $$\sum_{i\in I}t_{idj}{\hat{x}}_{bgidj}\leq T_{dj}\;\;\;\;\;\;\;\;\;\;\;\;\;\;\;\;\;\;\;\;\;\;\;\;\forall j\in J,d\in D,h\in H,g\in W:d>g$$

(46) $$\;\sum_{i\in I}t_{igj}{\hat{x}}_{bgigj}\leq T_{gj}+\Omega \;\;\;\;\;\;\;\;\;\;\;\;\;\;\;\;\;\;\;\;\;\;\;\;\forall j\in J,h\in H,g\in W$$

(47) $$\sum_{j\in J}x_{idj}+\sum_{j\in J}\sum_{k\in D:k<d}{\hat{x}}_{bgikj}\leq 1\;\;\;\;\;\;\;\;\;\;\;\;\;\;\;\;\;\;\;\;\;\;\;\;\forall i\in I,d\in D,b\in I,g\in W$$

(48) $$\sum_{j\in J}\sum_{k\in D}{\hat{x}}_{bgikj}\leq \sum_{j\in J}\sum_{d\in D}x_{idj}\;\;\;\;\;\;\;\;\;\;\;\;\;\;\;\;\;\;\;\;\;\;\;\;\forall g\in W,b\in I,i\in I$$

(49) $${\hat{x}}_{bgigj}\geq x_{igj}\;\;\;\;\;\;\;\;\;\;\;\;\;\;\;\;\;\;\;\;\;\;\;\;\forall b\in I,g\in W,i\in I,j\in J:i\neq b$$

Constraints (43)–(45) correspond to Constraints (4)–(6). Constraints (46) allow for some overtime to accommodate the substitute patient in a similar fashion to what was done in (36). Constraints (47) prevent patients’ admission dates in the reschedule being anticipated with respect to the original plan. Constraints (48) prevent patients who were not previously included in the NS being included in the new schedule. Lastly, Constraints (49) ensure that all patients included in the nominal schedule are included in the reschedule.

The model cannot be solved as a whole using an exact approach, except for some smaller instances. Furthermore, even rather small optimality gaps lead to solutions that are unacceptable for decision-makers, as the proposed plan overuses spare capacity to guarantee a feasible rescheduling for the days on which an RS is required. Consequently, we developed a heuristic approach that first computes an NS and then uses such schedule to compute a feasible solution for the overall problem applying:

1. A warm-start procedure;
2. An MILP-based sequential heuristic.

**Figure 1:**
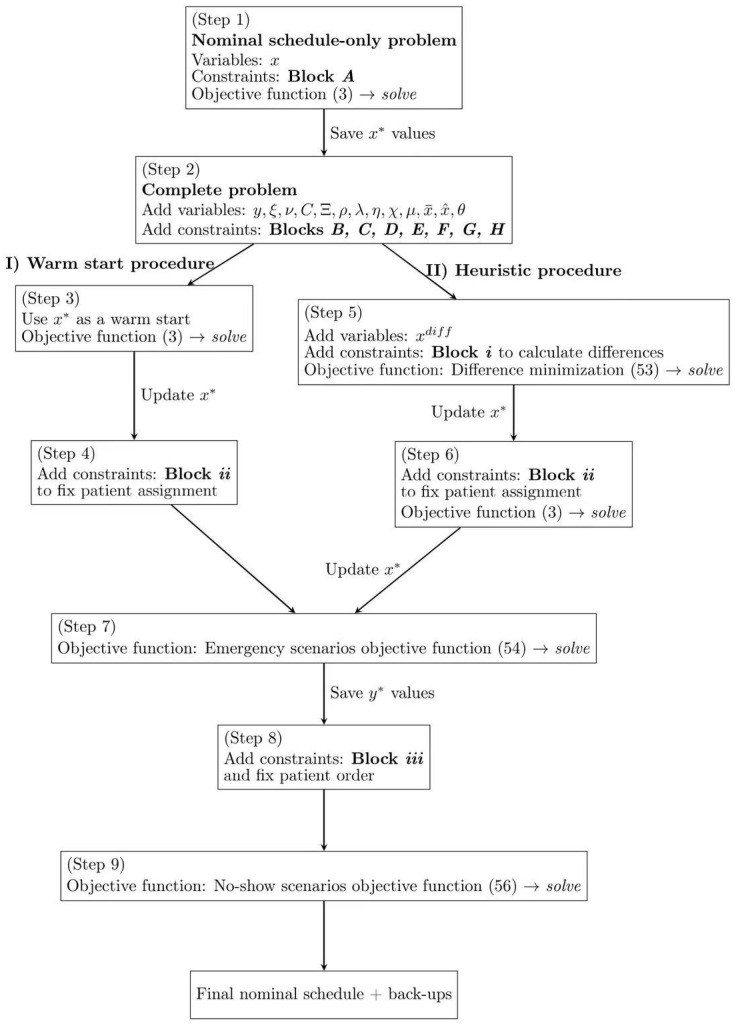
Schematic representation of the heuristic algorithm.

The RSs are then optimized based on patients’ penalties. Table 2 provides a summary of the parameters and decision variables used by the heuristic, while Figure 1 represents graphically the proposed approach. Table 2 contains all entities referenced in Equations (50)–(56) not introduced previously in Table 1.

In detail, we first solve the nominal problem, defined by Block A and objective function (3) (Step 1). The solution of this problem is stored in parameter $x_{idj}^{*}$. Then, the complete problem (Step 2) is considered and the warm start or the heuristic are applied to obtain a feasible complete solution.

The warm start computes a feasible solution of the complete problem (Step 3), whose assignments are then (Step 4) set through the following additional constraints (Block ii):

(50) $$\sum_{j\in J}x_{igj}\geq \sum_{j\in J}x_{igj}^{*}\;\forall i\in I,g\in W$$

Constraints (50) fix the admission dates of the patients assigned in the first |W| days of the NS of the warm-start procedure.

The heuristic procedure introduces the variable $x_{id}^{diff}$ to represent the difference between the NS (a Block A-only problem) and any feasible solution of the complete one (Blocks A, B, C, D, E, F, G, and H). The differences are computed through the following Block (i) of constraints:

(51) $$\sum_{j\in J}x_{idj}-\sum_{j\in J}x_{idj}^{*}\leq x_{id}^{diff}\;\;\;\;\;\;\;\;\;\;\;\;\;\;\;\;\;\;\;\;\;\;\;\;\forall i\in I,d\in D$$

(52) $$\sum_{j\in J}x_{idj}^{*}-\sum_{j\in J}x_{idj}\leq x_{id}^{diff}\;\;\;\;\;\;\;\;\;\;\;\;\;\;\;\;\;\;\;\;\;\;\;\;\forall i\in I,d\in D$$

In Step 5, a new problem is considered. It includes all the constraints of Blocks A–H and the additional constraints of Block i. The goal is to minimize the difference between the two solutions with the following objective function:

(53) $$min\sum_{i\in I}\sum_{d\in D}x_{id}^{diff}$$

The solution of this new complete schedule is stored, updating the parameter $x_{idj}^{*}$. Block ii of constraints is added and the problem is solved once more (Step 6) with the objective function (3). Here, the greater than or equal to sign of (50) allows the model to try and find solutions in which, at the cost of introducing differences with respect to the initial solution, OR capacity is better exploited. The value of $x_{igj}^{*}$ is updated once again (Step 6).

After having fixed a common starting point, the final NS, and regardless of whether it was obtained with the warm-start or the heuristic procedure, we can now optimize the back-up plans for each type of disruption. First, we optimize the emergency scenarios and then for the no-shows. This is a deliberate choice, because to better manage the emergencies it is necessary to establish the ordering, $y_{idjs}$, of patients, a decision that does not impact no-show management. To optimize for emergencies, we solve the complete model with the following objective function (Step 7), defined as a sum of penalties related to (not) assigning patients in emergency RSs, in a similar fashion to what was done in (3):

(54) $$min\frac{1}{\left|H|\cdot |W|\cdot |L\right|}\sum_{i\in I}\sum_{g\in W}\sum_{h\in H}\sum_{j\in J}\left\{\sum_{d\in D:d>g}p_{id}{\bar{x}}_{hgidj}+q_{i}\sum_{d\in D}\left(1-{\bar{x}}_{hgidj}\right)\right\}$$

As previously mentioned, the outcome of Step 7 also orders patients within their assigned days, a decision stored in parameter $y_{igjs}^{*}$. Block iii fixes the order and the OR assignment of patients (Step 8) with the following additional constraints:

(55) $$y_{igjs}\geq y_{igjs}^{*}\;\;\;\;\;\;\;\;\;\;\;\;\;\;\;\;\;\;\;\;\;\;\;\;\forall i\in I,g\in W,j\in J,s\in S$$

To optimize for no-shows, we solve the complete model with the following objective function (Step 9):

(56) $$min\frac{1}{\left|I|/|D\right|}\sum_{i\in I}\sum_{g\in W}\sum_{b\in I}\sum_{j\in J}\left\{\sum_{d\in D}p_{id}{\hat{x}}_{bgidj}+q_{i}\sum_{d\in D}\left(1-{\hat{x}}_{bgidj}\right)\right\}$$

**Figure 2:**
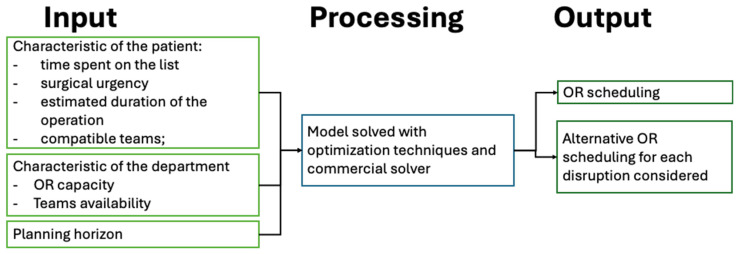
Scheme of the optimization tool.

A simplified scheme of the optimization tool is reported in Figure 2.

## 4. Results

**Figure 3:**
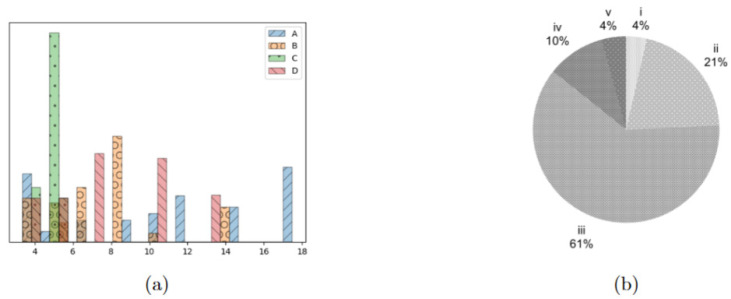
Characterization of the patients in the test instances. (**a**) distribution of surgical times for the four patient groups, expressed as multiples of 15 min. (**b**) Distribution of the different urgency classes in the tested instances, i being the most urgent and v the least one.

We first tested our approaches on a set of instances, based on [21]. In these instances, patients belong to one of five urgency classes, as proposed in [7], which are true to the Italian guidelines on surgical priorities. In the instances, |D| is equal to [14,28],|J|=[2,3],|W|=1 for all combinations. $a_{idj}$ was always set to 1, with the exception of days indexed by a d multiple of 6 or 7, which modeled the weekends. The time discretization (set H) is expressed in terms of 15 min blocks: as we considered 6 h of OR activity, we set $T_{dj}=24$; as one hour of overtime can be accepted for rescheduling, Ω was set to 4, while Δ and $\hat{\Delta }$ were set to 7 and 2, respectively, meaning that no-show patients must be rescheduled in two days, and patients cancelled because of an emergency must be rescheduled within a week. Lastly, the possible duration of emergencies was defined as $\gamma_{l}=[4,8,16]$. We considered instances with 40, 80, and 120 patients. For each cardinality of I, we considered four distinct patient populations, each characterized by a different patient mix in terms of surgical length, yielding four types of instances, A, B, C and D, as shown in Figure 3a, while Figure 3b reports the distribution of the urgency classes in all the tested instances, which was the same for all groups of patients. Approaches and models were implemented in gurobipy (for further information, see: pypi.org/project/gurobipy, accessed: 21 March 2024) and solved using Gurobi 11.0.1 (Gurobi Optimization LLC, Beaverton, OR, USA) in a jupyter environment. Experiments were run on a Dell Inspiron 15 5510 (Dell Inc., Austin, TX, USA) machine equipped with Microsoft Windows 11(Microsoft Corp., Redmond, WA, USA) and an Intel ^®^ Core ^TM^ i51135G7 CPU (Intel Corp., Santa Clara, CA, USA) and 16 GB of installed RAM. For all steps of the approach, a time limit of 900 s was imposed, without enforcing any memory limit. We compared the results of the heuristic procedure with those obtained with the warm-start procedure. We used the lower bound of the optimal solution of the overall problem as a reference to evaluate the quality of the obtained schedules with respect to the selected objective function. The results are reported in Table 3, Table 4 and Table 5.

The tables are structured as follows: the ID column reports the ID of the tested patient list. This, along with columns |D| and |J|, defines the tested instance. OF is the objective function of the nominal schedule problem (3), while $OF_{WS}$ and $LB_{WS}$ represent the objective function value of the complete problem (as calculated by the warm-start procedure) and its lower bound, respectively. Similarly, $OF_{Heu}$ is the objective function value of the problem calculated by the heuristic procedure. Columns $OF_{WS}-OF$ and $OF_{Heu}-OF$ calculate the difference between the two objective functions of the complete problem and that of the NS. This provides an indication of the price, in terms of OF value, of introducing a number of constraints to guarantee that a feasible RS exists for each disruption. Lastly, columns $\frac{OF_{WS}-LB_{WS}}{LB_{WS}}$ and $\frac{OF_{Heu}-LB_{WS}}{LB_{WS}}$ report the percentage gap between the objective function values computed by the warm-start and the heuristic procedure and the found lower bound.

In all tested instances, the NS problem (Step 1) was solved to optimality in an average of 21.5 s, except for three instances. In these, the solver reached the time limit, with an average gap of 0.02%. Including those three instances, all with cardinality |I|=120, the average computational time to solve the nominal problem was 76.4 s. Still considering all instances, the warm-start procedure (Step 3) always reaches the time limit with an average gap of 7.9% (maximum 135.8%, minimum 0.07%), while the heuristic procedure takes on average 732.7 s to provide a solution. The first part of the heuristic (Step 5) is solved to optimality in 27 instances out of 48.

The results for |I|=40 are reported in Table 3. The warm-start procedure improves with respect to the heuristic procedure in 11 instances out of 16, with an average gap from the lower bound of the complete problem of 3.72% versus an average gap of 3.99% reached by the heuristic. The difference between the two approaches is small, and judging them based on these first results only would not establish a clear winner.

For |I|=80, the warm-start procedure still performs better than the heuristic one in 11 cases out of 16, but the average gap with respect to the lower bound of the complete problem is higher for the warm start (4.97%) than for the heuristic (4.05%). This is due to the fact that the highest gaps of the warm start are larger than those of the heuristic. For instance, the maximum gap for the warm start is 17.97% versus a maximum of 8.95% (in different instances) for the heuristic. This superiority is also evident when looking at the average values of the prices of the robustness columns $\left(OF_{WS}-OF\right.$ and $OF_{Heu}-OF$): on average, the heuristic produces solutions with smaller increases in the objective function.

For |I|=120, the warm-start procedure performs better than the heuristic one only in 5 cases out of 16.

As the cardinality of |I| gets bigger, the heuristic procedure performs better than the warm start in most cases, and, most importantly, does not produce extremely poor results, something that the warm start is not capable of guaranteeing. For instance, in the case of B-type patients with |J|=3 and |D|=28, the gap between the objective function of the solution of the warm start and its lower bound exceeds 100%. On the other hand, the gap of the heuristic w.r.t. the lower bound has an average value of 2.7%.

Optimizing the emergency (Step 7) and no-show (Step 9) RSs turns out to be easy, regardless of starting from the solution obtained with the warm-start or the heuristic procedure. In fact, the average final gap obtained by solving the problem with objective functions (54) and (56) is always very small (below 1%), except for a single instance where it rises to more than 50%. This is the same instance (group D with |I|=120,|J|=3 and |D|=28) in which the gap of the warm-start solution exceeded 100%.

As for computational results, to generate a feasible NS and to prepare a complete set of back-up scenarios and RSs takes an average total of 984.3 s with the heuristic procedure and 1307.6 s with the warm start. The heuristic appears to be slightly faster than the warm start. On average, starting from the heuristic solution generates better values for objective functions (54) and (56) at the end of Step 7 and Step 9, respectively.

This is due to the fact that the heuristic provides, in general, a schedule closer to the nominal one, while the warm start, in the more challenging instances, may generate solutions with more spare capacity. This is especially true for instances with a bigger |I| cardinality.

To better understand why a warm-start or an heuristic approach is required, we also ran the full scheduling model—OF (3), Constraints (4)–(6) and (8)–(49)—using the A instances of |I|=[40,80,120], with a 14-day planning horizon and two ORs, the scenario that was most consistently the easiest one to solve. For |I|=40, the model reached the time limit with a gap of 0.57%, providing a satisfactory NS. For |I|=80, the model reached the time limit with a relatively small optimality gap (2.51%). However, upon closer inspection of the solution, the schedule showed slight under-utilization of one of the two ORs (a residual occupancy of almost 2 h, in which time several patients on the waiting list could potentially have been fit in). For |I|=120, the model reached the time limit with a gap of 7.48%. In the proposed solution, one of the two ORs was empty, and the other one only had one patient assigned to it (an occupation rate of ∼14%). This means that even if the full model is indeed capable of providing a solution for each considered cardinality, it can often be practically unacceptable.

### Case Study

**Figure 4:**
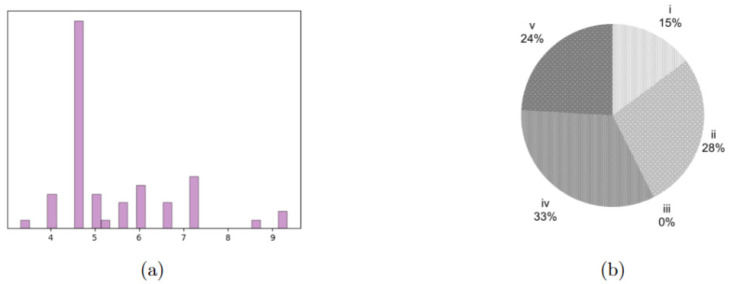
Characterization of the case study patients. (**a**) distribution of surgical times for the four patient groups, expressed as multiples of 15 min. (**b**) Distribution of the different urgency classes in the tested instances, i being the most urgent and v the least one.

V. Buzzi Children’s Hospital in Milan currently has two ORs that, as described at the beginning of this manuscript, are also devoted to hosting any emergency that may occur. They are, however, planning to build an additional one, so, also in this case, the tested instances consider cases with |J|=[2,3]. Similarly, the considered time horizon is |D|=[14,28], resulting in four instances. All other parameters were set as in the previous section. The realistic instance includes |I|=54 “ideal” DH patients, synthetically derived from examples of pathologies that typically fall within the DH procedures performed in the hospital. Their surgical times, reported in aggregate form in Figure 4a, represent therefore a realistic mix for this case study. Table 6 reports the objective functions and gaps for Steps 1, 3 and 6. The NS problem is solved to optimality for all four instances in an average computational time of 0.42 s. The warm-start procedure always reaches the time limit, and the heuristic procedure does so in two out of four instances, where three ORs are considered. The results reported in Table 6 show that, as expected, the warm start performs better than the heuristic, given that the number of patients is closer to that of the already tested instances with |I|=40. Still, it is worth highlighting that the warm start performs far better than the heuristic. This could be due to the different distribution of urgency classes in this case study, which has more very urgent patients, that individually impact the OF more.

In Table 7 we report the results associated with the RSs, that is, the values of (54) and (56): the warm start performs better when two ORs are considered, while the heuristic provides better results when three ORs are considered.

**Figure 5:**
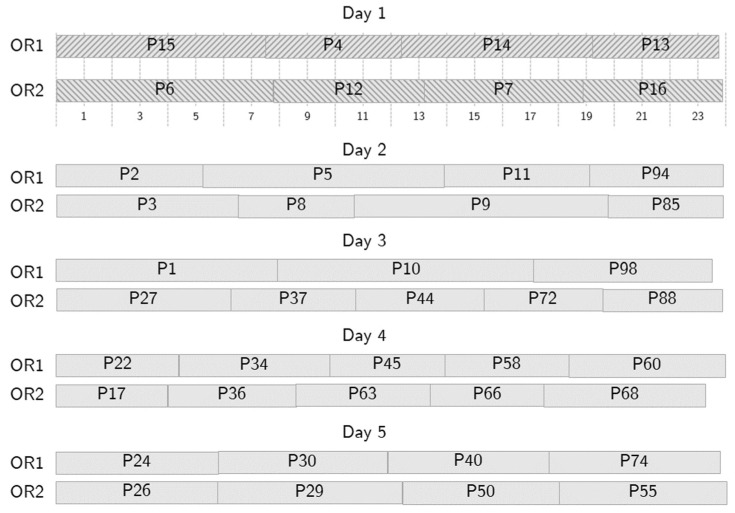
A sample nominal schedule.

**Figure 6:**
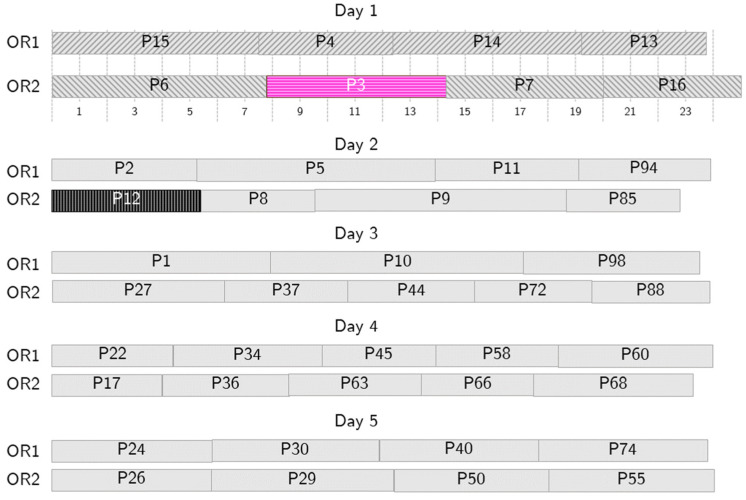
Sample behavior in case of no-show. Patient 12 (P12, in black) cannot undergo surgery on Day 1, so P3 (in pink) is called in as a substitute patient.

**Figure 7:**
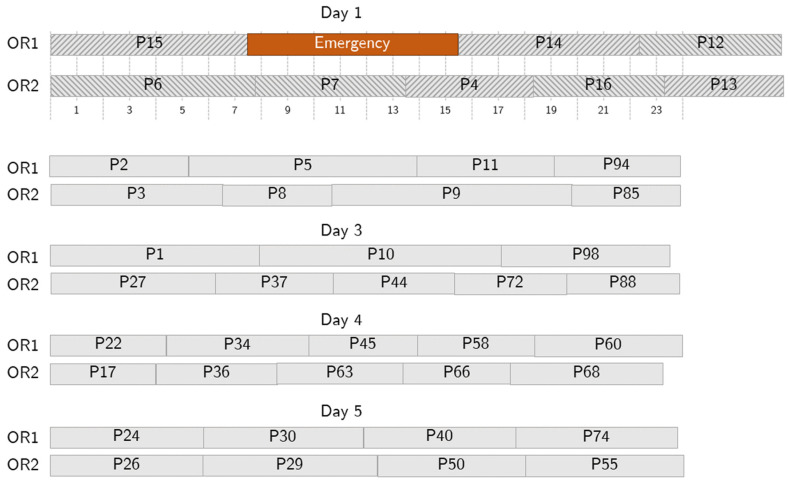
Sample behavior in case of a short emergency. The emergency (in orange) takes up some surgical time on Day 1, but no further action is needed because the allowed overtime is enough to accommodate it.

**Figure 8:**
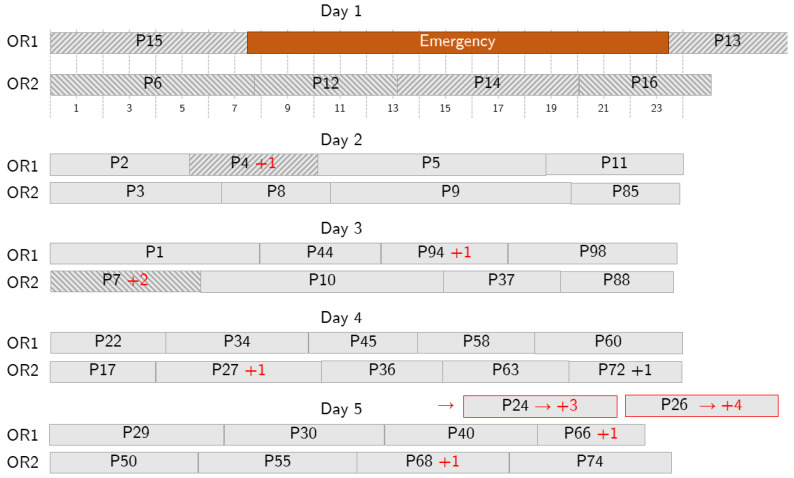
Sample behavior in case of a long emergency. The emergency (in orange) occupies some surgical time on Day 1 and further action is needed because the allowed overtime is not enough to accommodate it, so some patients are postponed. Delays are shown with red text. Red borders denote patients that are pushed out the depicted planning period.

To illustrate the behavior of the optimization tool, let us consider an example where patients are scheduled over 5 days. Figure 5, Figure 6, Figure 7 and Figure 8 show a partial output of the optimization tool. Figure 4 shows the NS, where 42 patients are scheduled in two ORs over five days. In the Figure, for each day, the patients scheduled in OR1 are shown above, while those scheduled in OR2 are shown below, with textured background to distinguish them from patients scheduled in following days. The starting time of each surgery is shown as well: the time horizon is divided into time slots, each fifteen minutes long, as shown below the schedule for OR1, and the 24 time slots represent six hours. So, for instance, patient number 15 is the first one on day one and requires almost two hours. Due to the requirements, no overtime is needed in the NS. The optimization tool generates an alternative schedule for all the possible no-shows on day one, that is, one for each patient scheduled on day one.

Figure 6 shows the RS to follow if patient 12, scheduled as second on day one in OR2, cannot be operated on. If such a no-show scenario occurs, the selected surrogate patient, patient 3, is summoned and undergoes surgery on day 1, swapping the position with patient 12. A short overtime makes this RS acceptable, and, as the nominal and the RS schedules are generated simultaneously, the swap can be implemented immediately, so that no vacant time, or, at least, a very short one, is suffered. Similar RSs are generated for each patient scheduled on day one and are immediately available.

Figure 7 shows an example of an RS employed to recover from an emergency. Here, we consider a short-length emergency occurring 1 h and 45 min after the starting time on day one. According to the RS, the emergency is assigned to OR1, the first OR available based on the NS, and patients 4 and 13 are swapped with patient 12 and moved to OR2. The optimization tool accepts one-hour overtime, and no further changes are needed. As for the no-show case, the RS is generated together with the NS, and the disruption is immediately recovered.

Figure 8 shows a case where a long-length emergency occurs and more changes are needed. The emergency is assigned to OR1 and impacts patients 4 and 14. Patient 14 is moved to OR2, and patient 7 is postponed to day 3 to make room for 14. Patient 4 is postponed to day 2. To make room for them, the schedules on the following days are reorganized: some of the patients are postponed (e.g., 94 to day 3 and 27 to day 4) and some are moved from one OR to another (e.g., patient 44 on day 3). However, the optimization tool operates to keep the number of changes limited, especially in the first days, and to reschedule all the patients within a given number of days.

## 5. Discussion

The OR is a huge resource for a hospital, often constituting the main source of income for the institution. However, there are some critical issues that can make its organization very difficult. Some of these critical issues are well known in both pediatric hospitals and university hospitals:

- The high volume of surgical specialties dedicated to the care of children in a single high-level center often determines the impossibility of having an OR specifically dedicated to emergencies. When an emergency happens, it is necessary to interrupt the activity in ORs in which scheduled procedures are in progress, to the detriment of the NS.
- In the pediatric field, there are more numerous and less predictable causes of cancellation/postponement of an intervention, such as fever, upper respiratory tract diseases, exanthematous diseases, and variables associated with fragile or syndromic patients or related to congenital malformations.
- Costs are generally higher, thus driving the need for efficiency: pediatric instrumentation can be more expensive and diagnosis-related groups (DRGs) less appropriate because both are often derived from the adult surgery practice and may not be very adaptable to pediatric patients.
- The allocation of resources in a TH must consider not only the care of patients, but also the training of students and residents, the collection of data for research purposes, and the definition and verification of new pathways and technologies.

Therefore, a pediatric TH can easily exceed the 5% limit of elective surgery cancellations on the scheduled day that is internationally recognized as reasonable for OR scheduling in the pediatric setting. This creates interruptions in surgical activities, making it more difficult to optimize resources, leading to significant economic losses and discomfort for patients, families, and healthcare personnel, endangering the sustainability of the system.

Currently, there are no standard tools to manage interruptions in surgical activities in the pediatric field. We thus propose a possible approach though an ILP-based model that, starting from certain specific assumptions about the TH pediatric environment for DH procedures, would allow us to optimize nominal programming and also to promptly resolve any unforeseen events coming from two main areas: emergencies, which interrupt scheduled activities, and last-minute cancellations, which lead to losses of operating spaces and economic resources. This work addressed multiple aspects of DH programming to:

- Efficiently manage interruptions leading to discontinuities in the elective procedures, promoting the prompt management of emergency cases;
- Optimize OR activities in case of elective surgery cancellations;
- Reduce the costs associated with both types of discontinuity;
- Better manage the time patients wait for surgery, equitably weighing urgency and time spent on the waiting list.

The model provides an automatic surgical scheduling tool for pediatric departments. The recovery programs are designed to minimize variations from the NS and to ensure that patients assigned to the first days in the planning horizon and whose operations are canceled due to emergencies are operated on within a limited number of days. Similarly, patients who do not show up must be rescheduled within a limited number of days. In all recovery programs, limited overtime for OR staff is accepted to reduce the inconvenience and overall costs of the episodes of interruption of the program.

The mathematical model generates in advance a response to all the expected reasons for disruption, providing the NS and RSs at the same time, ensuring a preventive solution to any discontinuity. This design therefore provides an immediate response to unforeseen events.

The model is intended to facilitate and speed up planning, which is largely delegated to the model itself: as RS creation is done when preparing the NS, no manual intervention or tuning is required in case of adverse events.

The choice of focusing on DH, which is a clinical approach that is increasingly relevant in the pediatric environment, is linked to the fact that the programming of interventions in DH is what most affects the waiting list. At the same time, since the interventions are generally of limited duration, it is easier to relocate them in the event of disruption; it is also easier to interrupt a DH OR in the event of an emergency. However, since the approach is versatile and can consider different operating times, it is foreseeable that it can also be adapted to different needs, such as the scheduling of ORs with non-DH types of surgery.

By choice, the model does not take into consideration some practical issues. One is the issue of hospital beds, which also entail problems of cancellation of scheduled surgical procedures. Another issue is the type of operator (expert, fellow, or resident), understood as the operating time necessary for the surgeon to complete the procedure. This does not prevent, however, these variables being inserted later. The mathematical model is in fact a preliminary model, subject to modifications, and has proven to be very manageable and ready to accommodate additional variables that characterize pediatric surgical activity in a TH.

The hypothesized possible advantages that the application of the model could offer would be: optimization of OR spaces; progressive shortening of surgical waiting lists thanks to a better use of operating spaces; automation of the scheduling process; greater satisfaction not only of administrative and OR staff, but also of patients and their families; and last but not least, significant economic savings for institutions.

Within the Italian national healthcare system, calculating the actual cost of an hour of OR activity is complex. Not only does it include medical and nursing staff wages, materials, anesthesia, instruments, and infrastructure, there is also no “official hourly price”. Further, in the context of a TH, the cost of mentoring is almost impossible to price. To provide a back-of-the-envelope benchmark, we can refer to the fact that it has been estimated that an hour of OR activity in a private Italian hospital would cost approximately €1200 (for further information, see https://www.anaao.it/content.php?cont=38216 (in Italian, accessed: 15 January 2026)). The calculation, however, is more complex and must also consider the value assigned to each procedure by the DRG table. These costs and considerations apply equally to both OR hours lost, for example, because of no-shows, and overtime hours required in the event of emergencies or delays in scheduled procedures.

The main limitation of this work is the lack of benchmarking with existing state-of-the-art techniques or even a quantitative baseline. First, a direct comparison with state-of-the-art techniques is unfeasible, as currently there is no standard-use model specifically designed to manage disruptions in pediatric operating room scheduling. As illustrated in Section 2, existing approaches in the literature predominantly address adult settings and/or treat emergencies and no-shows by means of online rescheduling procedures, or by using stochastic or robust programming techniques, which are based on assumptions substantially different from those of our work. In contrast, our proposed ILP-based framework is specifically designed to simultaneously generate an NS and a structured set of RSs in advance on the basis of representative scenarios. Therefore, the absence of a direct benchmarking comparison is linked to the novelty of the contribution: with this work, we introduce a novel decision-support paradigm for the pediatric DH environment rather than proposing an improvement/alternative to an existing approach defined under comparable assumptions.

Concerning the lack of a quantitative baseline, with this study it was only possible to acquire data from an internal survey (unpublished) on the number of patients whose surgeries were postponed in the 24 h before their scheduled date and time, in an observation period from March 2024 to March 2025, at the V. Buzzi Children’s Hospital. Out of a total of 1086 scheduled procedures, 178 (16.4%) were canceled in the preceding 24 h. Of these, the large majority (141, almost 80% of the total) were DH procedures. Considering 48 weeks of surgical activity during the same year, the number of postponed procedures was 2.9 per week, for a rough estimate of lost operating room time of approximately 4.5 h on a weekly basis. These data report a rough estimate of the total effect of disruptions.

Our model is still theoretical, and real-life experimentation in pediatric surgery scheduling must be started. In view of this, we have here demonstrated that the direction is promising.

## 6. Conclusions

We considered the problem of scheduling elective surgeries in a teaching pediatric hospital, also addressing the possible eventualities that disrupt the daily programming, using a mathematical model. We formulated the problem of defining an NS and a set of RSs, one for each considered disruption scenario as a disruption/restoration-based MILP model. Solving the model with an exact approach does not provide acceptable solutions, even with small optimality gaps, so we implemented a warm-start procedure and a heuristic procedure to provide better solutions to the problem. The warm-start procedure performs better than the heuristic one in smaller instances but becomes unstable in bigger ones. Nevertheless, both proposed approaches are able to solve the problem in a reasonable time. The model has proven to be able to address the problem of nominal programming and the prediction of alternative scenarios in case of same-day cancellations and/or the arrival of emergencies that interrupt the scheduled activity. The approaches offer the great advantage of providing a set of RSs that are ready to be deployed immediately; however, in this work we did not consider any downstream resources: this represents a limitation on the applicability of this work that will be explored in future research. The future prospect is to test it in the reality of the programming of a teaching pediatric hospital.

## Figures and Tables

**Table 1:** Summary of all sets, parameters and decision variables used in the MILP model.

Sets	
I	Patients on the waiting list
J	ORs
D	Days (indexed by d) of the planning horizon
W	Days (indexed by g) on which we want to create RSs
S	Position in the daily plan occupied by a patient
H	Time-slot discretization of the duration of the day; starts at 0
L	Set of possible types of emergency lengths
Parameters	
$a_{idj}$	Compatibility matrix for patient i, day d and OR j
$m_{i}$	Days spent on the waiting list by patient i
$u_{i}$	Priority class of patient i
$l_{i}$	Surgery deadline of patient i
$q_{i}$	Penalty for not assigning patient i
$p_{id}$	Penalty for assigning patient i to day d
$T_{dj}$	Availability of OR j on day d, expressed in time slots
$t_{idj}$	Duration of i’s surgery on day d and in OR j, expressed in time slots
$\gamma_{l}$	Duration of emergency in scenario l
Ω	Acceptable overtime in case of a substitution of a no-show or an emergency, expressed in time slots
$\hat{\Delta }$	Fixed delay used to reschedule no-show patients, expressed in days
Δ	Maximum rescheduling delay
Decision variables	
$x_{idj}$	Binary, assignment of patient i to day d and OR j
$y_{igjs}$	Binary, assignment of patient i on day g to OR j to slot s (ordering)
$\nu_{irgj}$	Binary, equal to 1 if patient i is assigned before patient r on day g and to OR j
$\xi_{igj}$	Continuous, non-negative expected starting time (in time slots) of the surgery for i on g,j
$C_{gj}$	Continuous, non-negative end time of last patient in room j on day g
$\rho_{hgj}$	Binary, equal to 1 if room j is empty when an emergency in time slot h of day g occurs
$\lambda_{igjh}$	Binary, equal to 1 if patient i is currently being operated on in OR j when an emergency occurs in time slot h of day g
$\Xi_{hgj}$	Continuous, non-negative first availability of room j to be assigned for emergency occurring in time slot h of day g
$\eta_{hgj}$	Binary, equal to 1 if an emergency occurring in time slot h of day g is assigned to j
$\chi_{hgi}$	Binary, equal to 1 if the assignment of i is affected by an emergency occurring in time slot h of day g
${\bar{x}}_{\mathrm{hglidj}\;}$	Binary, equal to 1 if i needs to be rescheduled on day d and to OR j because of emergency occurring in time slot h of day g
$\mu_{hgli}$	Binary, equal to 1 if patient i cannot be rescheduled if an emergency of type l occurs in time slot h of day g
${\hat{x}}_{bgidj}$	Binary, equal to 1 if i needs to be rescheduled on day d and to OR j because of a no-show patient, b, on day g
$\theta_{igj}$	Binary, equal to 1 if patient i is the substitute patient for day g and OR j in case of no-shows

**Table 2:** Parameters and decision variables used in heuristic algorithm.

Parameters	
$x_{idj}^{*}$	Binary parameter storing the NS solution
$y_{igjs}^{*}$	Binary parameter storing the order of the NS solution
Decision variables	
$x_{id}^{\mathrm{diff}\;}$	Binary, equal to 1 if the assignment of patient i on day d differs from the nominal-only solution

**Table 3:** Results for |I|=40.

ID	|D|	|J|	OF	$LB_{WS}$	$OF_{WS}$	$OF_{Heu}$	$OF_{WS}-OF$	$OF_{Heu}-OF$	$\frac{OF_{WS}-LB_{WS}}{LB_{WS}}$	$\frac{OF_{Heu}-LB_{WS}}{LB_{WS}}$
40 A	14	2	4891	4886	4913	4939	22	48	0.55%	1.08%
40 A	14	3	4584	4579	4611	4668	27	84	0.70%	1.94%
40 A	28	2	5067	5059	5096	5151	29	84	0.73%	1.82%
40 A	28	3	4659	4651	4783	4707	124	48	2.84%	1.20%
40 B	14	2	3200	3200	3223	3387	23	187	0.72%	5.84%
40 B	14	3	3004	3003	3332	3202	328	198	10.96%	6.63%
40 B	28	2	3289	3286	3323	3938	34	649	1.13%	19.84%
40 B	28	3	3033	3028	3689	3348	656	315	21.83%	10.57%
40 C	14	2	3138	3138	3156	3166	18	28	0.57%	0.89%
40 C	14	3	3005	3005	3015	3045	10	40	0.33%	1.33%
40 C	28	2	3214	3214	3232	3286	18	72	0.56%	2.24%
40 C	28	3	3024	3024	3300	3060	276	36	9.13%	1.19%
40 D	14	2	5768	5768	5773	5852	5	84	0.09%	1.46%
40 D	14	3	5486	5481	5506	5638	20	152	0.46%	2.86%
40 D	28	2	5863	5863	5899	5971	36	108	0.61%	1.84%
40 D	28	3	5523	5522	5978	5691	455	168	8.26%	3.06%
Average			4172	4169	4302	4316	130	144	3.72%	3.99%

**Table 4:** Results for |I|=80.

ID	|D|	|J|	OF	$LB_{WS}$	$OF_{WS}$	$OF_{\mathrm{Heu}\;}$	$OF_{WS}-OF$	$OF_{Heu}-OF$	$\frac{OF_{WS}-LB_{WS}}{LB_{WS}}$	$\frac{OF_{Heu}-LB_{WS}}{LB_{WS}}$
80 A	14	2	9677	9677	9716	9886	39	209	0.40%	2.16%
80 A	14	3	8888	8881	8965	9383	77	495	0.95%	5.65%
80 A	28	2	10,631	10,629	10,682	10,807	51	176	0.50%	1.67%
80 A	28	3	9428	9420	10,310	9550	882	122	9.45%	1.38%
80 B	14	2	8947	8939	9086	9156	139	209	1.64%	2.43%
80 B	14	3	8315	8295	9219	8507	904	192	11.14%	2.56%
80 B	28	2	9494	9485	9602	9784	108	290	1.23%	3.15%
80 B	28	3	8637	8615	9712	9052	1075	415	12.73%	5.07%
80 C	14	2	8429	8428	8498	8621	69	192	0.83%	2.29%
80 C	14	3	7845	7825	9231	7929	1386	84	17.97%	1.33%
80 C	28	2	8832	8823	8920	9048	88	216	1.10%	2.55%
80 C	28	3	8033	7957	8189	8669	156	636	2.92%	8.95%
80 D	14	2	9405	9394	9408	9828	3	423	0.15%	4.62%
80 D	14	3	8730	8713	8959	9121	229	391	2.82%	4.68%
80 D	28	2	10,184	10,176	10,523	10,514	339	330	3.41%	3.32%
80 D	28	3	9153	9112	10,226	10,296	1073	1143	12.23%	12.99%
Average			9039	9023	9453	9384	414	345	4.97%	4.05%

**Table 5:** Results for |I|=120.

ID	|D|	|J|	OF	$LB_{WS}$	$OF_{WS}$	$OF_{Heu}$	$OF_{WS}-OF$	$OF_{Heu}-OF$	$\frac{OF_{WS}-LB_{WS}}{LB_{WS}}$	$\frac{OF_{Heu}-LB_{WS}}{LB_{WS}}$
120 A	14	2	19,669	19,655	19,750	19,892	81	223	0.48%	1.21%
120 A	14	3	17,829	17,767	17,928	18,281	99	452	0.91%	2.89%
120 A	28	2	22,355	22,345	22,712	22,547	357	192	1.64%	0.90%
120 A	28	3	19,405	19,148	21,769	19,693	2364	288	13.69%	2.85%
120 B	14	2	17,958	17,948	18,097	18,186	139	228	0.83%	1.33%
120 B	14	3	16,516	16,428	20,386	17,125	3870	609	24.09%	4.24%
120 B	28	2	19,773	19,693	21,411	20,960	1638	1187	8.72%	6.43%
120 B	28	3	17,385	17,242	40,659	18,572	23,274	1187	135.81%	7.71%
120 C	14	2	16,801	16,792	16,907	17,412	106	611	0.68%	3.69%
120 C	14	3	15,478	15,408	19,313	15,838	3835	360	25.34%	2.79%
120 C	28	2	17,734	17,676	19,433	18,018	1699	284	9.94%	1.93%
120 C	28	3	16,033	15,865	16,347	16,357	314	324	3.04%	3.10%
120 D	14	2	19,100	19,087	19,100	19,100	0	0	0.07%	0.07%
120 D	14	3	17,401	17,334	17,626	17,587	225	186	1.68%	1.46%
120 D	28	2	21,604	21,527	21,604	21,604	0	0	0.36%	0.36%
120 D	28	3	18,769	18,613	20,931	19,036	2162	267	12.45%	2.27%
Average			18,363	18,283	20,873	18,763	2510	400	14.98%	2.70%

**Table 6:** Results for the realistic instance.

|D|	|J|	OF	$LB_{WS}$	$OF_{WS}$	$OF_{Heu}$	$OF_{WS}-OF$	$OF_{Heu}-OF$	$\frac{OF_{WS}-LB_{WS}}{LB_{WS}}$	$\frac{OF_{Heu}-LB_{WS}}{LB_{WS}}$
14	2	2268	2269	2278	2885	10	617	0.40%	27.15%
14	3	2106	2106	2488	2367	382	261	18.14%	12.39%
28	2	2400	2394	2453	3102	53	702	2.46%	29.57%
28	3	2146	2138	2359	2247	213	101	10.34%	5.10%
Average		2230	2227	2395	2650	165	420	7.83%	18.55%

**Table 7:** Results for emergency and no-show scenarios, comparing the average objective function values of the solutions obtained with the warm start and with the heuristic in the realistic instances.

|D|	|J|	$OF_{WS}^{\mathrm{emergency}\;}$	$OF_{Heu}^{\mathrm{emergency}\;}$	$OF_{WS}^{\mathrm{noshow}\;}$	$OF_{Heu}^{\mathrm{noshow}\;}$
14	2	2289.0	2891.5	2284.6	2862.7
14	3	2444.0	2367.2	2433.8	2354.3
28	2	2461.1	3107.2	2455.2	3105.6
28	3	2359.0	2247.0	2259.1	2251.6
Average		2388.3	2653.2	2358.2	2643.5

## Data Availability

The data reported in this study are available upon request from the corresponding author.

## References

[1] Pulido R., Aguirre A.M., Ortega-Mier M., García-Sánchez Á., Méndez C.A. (2014). Managing daily surgery schedules in a teaching hospital: A mixed-integer optimization approach. BMC Health Serv. Res..

[2] Eshghali M., Kannan D., Salmanzadeh-Meydani N., Esmaieeli Sikaroudi A.M. (2023). Machine learning based integrated scheduling and rescheduling for elective and emergency patients in the operating theatre. Ann. Oper. Res..

[3] Koh W.X., Phelan R., Hopman W.M., Engen D. (2021). Cancellation of elective surgery: Rates, reasons and effect on patient satisfaction. Can. J. Surg..

[4] Ben Mansour M., Lassioued O., Chakroun S., Slimene A., Ben Youssef S., Ksiaa A., Gahbiche M. (2023). Elective surgery cancellations in pediatric surgery: Rate and reasons. BMC Pediatr..

[5] Kaddoum R., Fadlallah R., Hitti E., El-Jardali F., El Eid G. (2016). Causes of cancellations on the day of surgery at a Tertiary Teaching Hospital. BMC Health Serv. Res..

[6] Broch A., Paye-Jaouen A., Bruneau B., Glenisson M., Taghavi K., Botto N., Goulin J., Lopez P., Querciagrossa S., El Ghoneimi A. (2023). Day Surgery in Children Undergoing Retroperitoneal Robot-assisted Laparoscopic Pyeloplasty: Is It Safe and Feasible?. Eur. Urol. Open Sci..

[7] Valente R., Testi A., Tanfani E., Fato M., Porro I., Santo M., Santori G., Torre G., Ansaldo G. (2009). A model to prioritize access to elective surgery on the base of clinical urgency and waiting time. BMC Health Serv. Res..

[8] Cardoen B., Demeulemeester E., Belien J. (2010). Operating room planning and scheduling: A literature review. Eur. J. Oper. Res..

[9] Guerriero F., Guido R. (2011). Operational research in the management of the operating theatre: A survey. Health Care Manag. Sci..

[10] Zhu S., Fan W., Yang S., Pei J., Pardalos P.M. (2018). Operating room planning and surgical case scheduling: A review of literature. J. Comb. Optim..

[11] Wang L., Demeulemeester E., Vansteenkiste N., Rademakers F.E. (2021). Operating room planning and scheduling for outpatients and inpatients: A review and future research. Oper. Res. Health Care.

[12] Blake J.T., Carter M.W. (2002). A goal programming approach to strategic resource allocation in acute care hospitals. Eur. J. Oper. Res..

[13] Dexter F., Ledolter J., Wachtel R.E. (2005). Tactical decision making for selective expansion of operating room resources incorporating financial criteria and uncertainty in subspecialties’ future workloads. Anesth. Analg..

[14] Banditori C., Cappanera P., Visintin F. (2013). A combined optimization–simulation approach to the master surgical scheduling problem. IMA J. Manag. Math..

[15] Erdogan S.A., Denton B.T. (2011). Surgery planning and scheduling. Encyclopedia of Operations Research and Management Science.

[16] Marques I., Captivo M.E., Barros N. (2019). Optimizing the master surgery schedule in a private hospital. Oper. Res. Health Care.

[17] Denton B., Viapiano J., Vogl A. (2007). Optimization of surgery sequencing and scheduling decisions under uncertainty. Health Care Manag. Sci..

[18] Batun S., Denton B., Huschka T., Schaefer A. (2011). Operating room pooling and parallel surgery processing under uncertainty. Inf. J. Comput..

[19] Clavel D., Mahulea C., Albareda J., Silva M. (2020). A Decision Support System for Elective Surgery Scheduling under Uncertain Durations. Appl. Sci..

[20] Shylo O., Prokopyev O., Schaefer A. (2013). Stochastic Operating Room Scheduling for High-Volume Specialties under Block Booking. Inf. J. Comput..

[21] Addis B., Carello G., Grosso A., Tànfani E. (2016). Operating Room Scheduling and Rescheduling: A Rolling Horizon Approach. Flex. Serv. Manuf. J..

[22] Addis B., Carello G., Tànfani E. (2024). Evaluating the Impact of the Level of Robustness in Operating Room Scheduling Problems. Healthcare.

[23] Wang Y., Zhang Y., Tang J. (2024). Wasserstein distributionally robust surgery scheduling with elective and emergency patients. Eur. J. Oper. Res..

[24] Tait A.R., Voepel-Lewis T., Munro H.M., Gutstein H.B., Reynolds P. (1997). Cancellation of pediatric outpatient surgery: Economic and emotional implications for patients and their families. J. Clin. Anesth..

[25] Pratap J., Varughese A.M., Mercurio P., Lynch T., Lonnemann T., Ellis A., Rugg J., Stone W.R., Bedinghaus C. (2015). Reducing cancelations on the day of scheduled surgery at a children’s hospital. Pediatrics.

[26] Cappanera P., Visintin F., Banditori C. (2018). Addressing conflicting stakeholders’ priorities in surgical scheduling by goal programming. Flex. Serv. Manuf. J..

[27] Aringhieri R., Bruni M.E., Khodaparasti S., van Essen J.T. (2017). Emergency medical services and beyond: Addressing new challenges through a wide literature review. Comput. Oper. Res..

[28] Duma D., Aringhieri R. (2019). The management of non-elective patients: Shared vs. dedicated policies. Omega.

[29] Rachuba S., Werners B. (2017). A fuzzy multi-criteria approach for robust operating room schedules. Ann. Oper. Res..

[30] Lamiri M., Xie X., Dolgui A., Grimaud F. (2008). A stochastic model for operating room planning with elective and emergency demand for surgery. Eur. J. Oper. Res..

[31] Essen J.T., Hans E.W., Hurink J.L., Oversberg A. (2012). Minimizing the waiting time for emergency surgery. Oper. Res. Health Care.

[32] Bargetto R., Garaix T., Xie X. (2018). Dynamic insertion of emergency surgeries with different waiting time targets. IEEE Trans. Autom. Sci. Eng..

[33] Miao H., Wang J.J. (2021). Scheduling elective and emergency surgeries at shared operating rooms with emergency uncertainty and waiting time limit. Comput. Indust Eng..

[34] Jha R.K., Gajpal Y., Chattopadhyay M., Yang X. (2023). Multiple operation theatre scheduling for mitigating the disturbance caused by emergency patients. Syst. Soft Comput..

[35] Boccia M., Mancuso A., Masone A., Sterle C. (2024). Integrated operating room planning and scheduling: An ILP-Based off-line approach for emergency responsiveness at a local hospital in Naples. Soft Comput..

[36] Dantas L.F., Fleck J.L., Cyrino Oliveira F.L., Hamacher S. (2018). No-shows in appointment scheduling–a systematic literature review. Health Policy.

[37] Zacharias C., Pinedo M. (2014). Appointment scheduling with no-shows and overbooking. Prod. Oper. Manag..

[38] Berg B.P., Denton B.T., Erdogan S.A., Rohleder T., Huschka T. (2014). Optimal booking and scheduling in outpatient procedure centers. Comput. Oper. Res..

[39] M’Hallah R., Visintin F. (2019). A stochastic model for scheduling elective surgeries in a cyclic master surgical schedule. Comput. Ind. Eng..

[40] Ozen A., Marmor Y., Rohleder T., Balasubramanian H., Huddleston J., Huddleston P. (2016). Optimization and simulation of orthopedic spine surgery cases at Mayo Clinic. Manuf. Serv. Oper. Manag..

[41] Salazar L.H.A., Leithardt V.R.Q., Parreira W.D., da Rocha Fernandes A.M., Victória Barbosa J.L., Duarte Correia S. (2021). Application of machine learning techniques to predict a patient’s no-show in the healthcare sector. Future Internet.

